# An assessment of adult mosquito collection techniques for studying species abundance and diversity in Maferinyah, Guinea

**DOI:** 10.1186/s13071-020-04023-3

**Published:** 2020-03-24

**Authors:** Cintia Cansado-Utrilla, Claire L. Jeffries, Mojca Kristan, Victor A. Brugman, Patrick Heard, Gnepou Camara, Moussa Sylla, Abdoul H. Beavogui, Louisa A. Messenger, Seth R. Irish, Thomas Walker

**Affiliations:** 1 Department of Disease Control, London School of Hygiene and Tropical Medicine, Keppel Street, London, WC1E 7HT UK; 2 Centre de Formation et de Recherche en Sante Rurale de Maferinyah, Conakry, Republic of Guinea; 3 Entomology Branch, Centers for Disease Control and Prevention, Atlanta, GA 30329-4027 USA; 4 American Society for Microbiology, 1752 N Street, NW, Washington, DC 20036 USA; 5 The US President’s Malaria Initiative and Entomology Branch, Centers for Disease Control and Prevention, Atlanta, GA 30329-4027 USA

**Keywords:** BG sentinel 2 trap, CDC light trap, Gravid Trap, Guinea, Mosquito, Stealth trap

## Abstract

**Background:**

Several mosquito collection methods are routinely used in vector control programmes. However, they target different behaviours causing bias in estimation of species diversity and abundance. Given the paucity of mosquito trap data in West Africa, we compared the performance of five trap-lure combinations and Human Landing Catches (HLCs) in Guinea.

**Methods:**

CDC light traps (LT), BG sentinel 2 traps (BG2T), gravid traps (GT) and Stealth traps (ST) were compared in a 5 × 5 Latin Square design in three villages in Guinea between June and July 2018. The ST, a portable trap which performs similarly to a LT but incorporates LEDs and incandescent light, was included since it has not been widely tested. BG2T were used with BG and MB5 lures instead of CO_2_ to test the efficacy of these attractants. HLCs were performed for 5 nights, but not as part of the Latin Square. A Generalised Linear Mixed Model was applied to compare the effect of the traps, sites and collection times on mosquito abundance. Species identification was confirmed using PCR-based analysis and Sanger sequencing.

**Results:**

A total of 10,610 mosquitoes were captured across five traps. ST collected significantly more mosquitoes (7096) than the rest of the traps, but resulted in a higher number of damaged specimens. ST and BG2T collected the highest numbers of *Anopheles gambiae* (*s.l*.) and *Aedes aegypti* mosquitoes, respectively. HLCs captured predominantly *An. coluzzii* (41%) and hybrids of *An. gambiae* and *An. coluzzii* (36%) in contrast to the five traps, which captured predominantly *An. melas* (83%). The rural site (Senguelen) presented the highest abundance of mosquitoes and overall diversity in comparison with Fandie (semi-rural) and Maferinyah Centre I (semi-urban). Our results confirm the presence of four species for the first time in Guinea.

**Conclusions:**

ST collected the highest number of mosquitoes suggesting this trap may play an important role for mosquito surveillance in Guinea and similar sites in West Africa. We recommend the incorporation of molecular tools in entomological studies since they have helped to identify 25 mosquito species in this area.
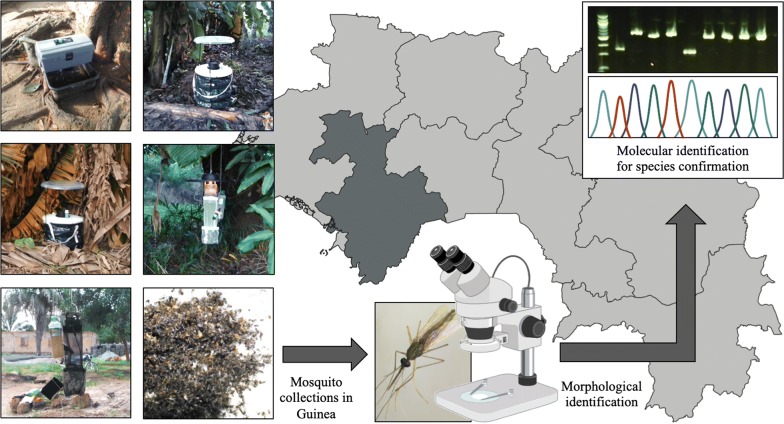

## Background

Control programmes which target malaria and other vector-borne diseases need to be specific to the country or region in which they are implemented. In order to choose the best intervention(s), it is essential to know which mosquito species are both present and transmitting human pathogens in a given area. For example, the primary vectors of malaria in Africa often display primarily endophagic and endophilic behaviour and therefore can be targeted by interventions such as indoor residual spraying (IRS) or through the use of long-lasting insecticidal nets (LLINs). Despite primary vectors contributing to the majority of the transmission of mosquito-borne diseases, secondary vector species can play an essential role in maintaining residual transmission [1], be less affected by interventions focused on primary vectors and increase in dominance and relative importance [2, 3]. Therefore, control programmes that do not target secondary vectors may have reduced success [4]. In order to monitor the effectiveness of a control programme, mosquito abundance and composition before and after intervention deployment can be determined by undertaking entomological surveys.

Different collection methods are available to collect entomological data, among which human landing catches (HLCs) are the gold standard method for collecting human-biting mosquito species [5]. However, HLCs only collect anthropophilic, host-seeking mosquito species. Therefore, additional methods of adult mosquito sampling can be used indoors and outdoors to exploit different aspects of mosquito feeding and resting behaviour including anthropophily, zoophily, endophily, exophily, endophagy and exophagy. However, trap comparison studies are necessary to determine trap efficacy given the variety of different mosquito species behaviours. Factors that can influence the abundance, species composition, female physiological status (gravid, blood-fed, etc.) and infection prevalence of the collection include trap design, use of attractants and location [6–8]. Therefore, it is important to consider trap bias to decide which one is most appropriate for mosquito monitoring and surveillance objectives in a given location. Although some traps have been compared to HLCs in East Africa [6], to our knowledge only a few studies have compared the performance of mosquito traps in West Africa (for example, in Ghana [9] and Senegal [8]). In Western Kenya, catches rates for *An. gambiae* (*s.l*.) were high for both CDC light traps (LT) and HLCs performed outdoors compared to HLCs performed indoors suggesting traps can play an important role in malaria entomological surveillance [6].

Guinea is a West African country with a high prevalence of vector-borne diseases [10, 11] where more than 55% of the population is affected by poverty [12]. Major outbreaks of human diseases include a yellow fever virus (YFV) outbreak in 2000 [13] where *Aedes aegypti*, the major YFV vector in urban areas, was not found in the rural areas [13], suggesting other mosquito species were likely involved in transmission. Despite significant transmission of malaria, lymphatic filariasis and sporadic outbreaks of arboviruses, relatively few medical entomological studies to date have been undertaken in Guinea [14–22]. Therefore, there is a need to undertake entomological surveys using diverse collection methods to determine the most appropriate mosquito trapping methods to use for surveillance.

We compared the performance of five adult trapping methods to determine mosquito species abundance and diversity in Maferinyah sub-prefecture, Guinea, and provide evidence towards the most suitable trap for surveillance. To our knowledge, only larval collections, pyrethroid spray catches, exit traps, aspirators, HLCs and LT have been used in Guinea to collect mosquitoes [16, 19, 22–24]. In this study, we selected gravid traps (GT), Stealth traps (ST), LT and BG sentinel 2 traps (BG2T) with two different lures (BG and MB5) in comparison with HLCs to test new trapping methods not previously used in Guinea to capture the highest diversity of mosquito species. The abundance and diversity of mosquito species captured was assessed in three locations presenting different geographical conditions, i.e. rural, semi-rural and semi-urban, and the results of this entomological survey are discussed in the context of mosquito surveillance and vector control strategies.

## Methods

### Study sites

**Fig. 1:**
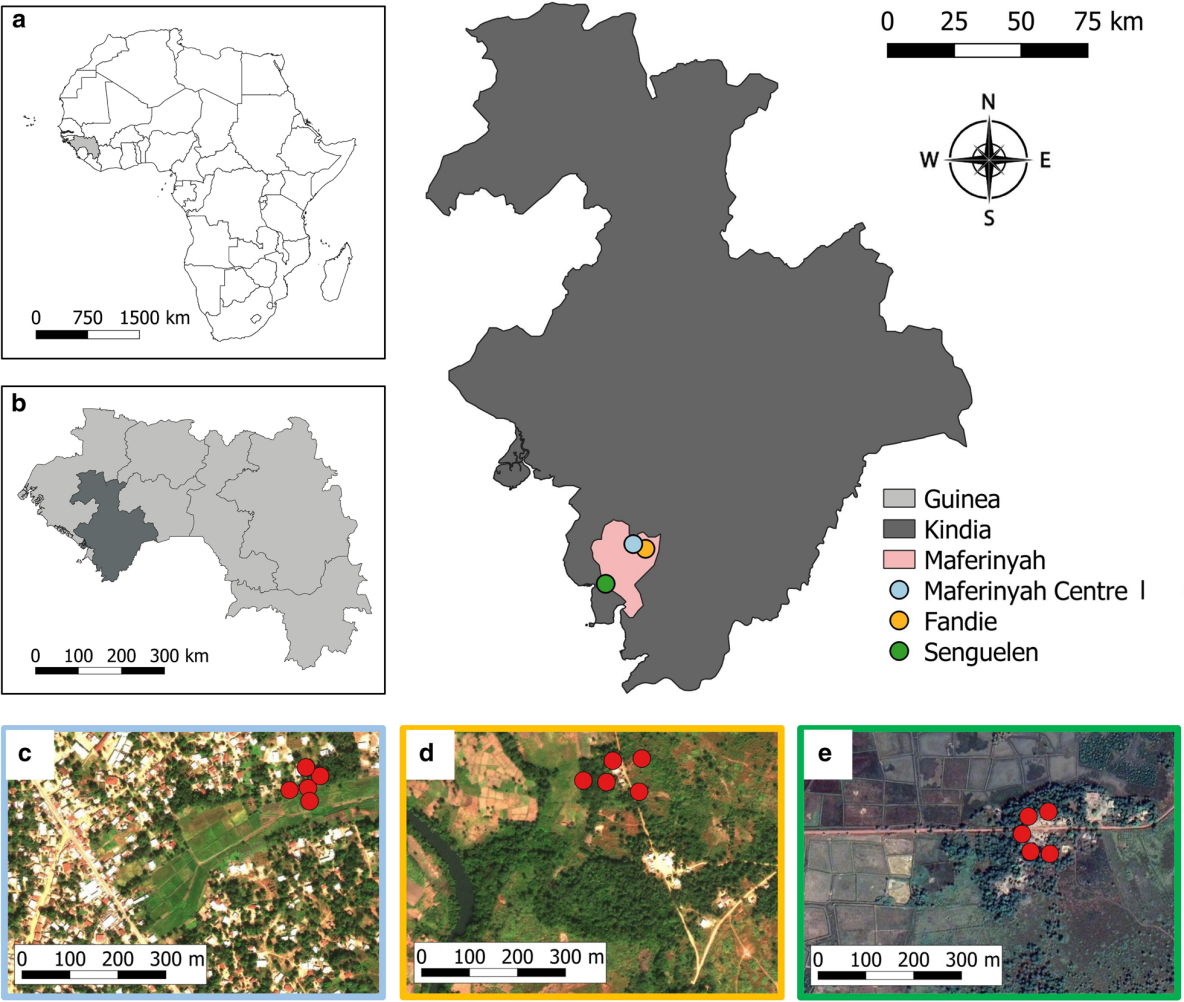
Location of the Maferinyah sub-prefecture and the three study sites in Kindia, Guinea, for the mosquito trap comparison. **a** Guinea (light grey) in Africa. **b** Region of Kindia (dark grey) in Guinea. **c** Sampling points (red) in Maferinyah Centre I. **d** Sampling points (red) in Fandie. **e** Sampling points (red) in Senguelen. Maps were obtained using QGIS. Basemaps were obtained from ArcGIS online and Google Maps Satellite

In order to compare mosquito diversity and determine the efficacy of different trap types between rural, semi-rural and semi-urban locations, three sites were selected for mosquito collections using traps: Senguelen, Fandie and Maferinyah Centre I, respectively (Fig. 1). The corresponding coordinates in decimal degrees of latitude and longitude are as follows: Senguelen (9.411, − 13.375), Fandie (9.53, − 13.239) and Maferinyah Centre I (9.546, − 13.281). HLCs were performed in Senguelen. All the study sites are located in the Maferinyah sub-prefecture, located within the Forecariah prefecture, in the Kindia region of Guinea. For the trap comparison, five sampling locations were chosen within each site, with a minimum of 50 m between each one. The coordinates of sampling locations were recorded using GPS (eTrex 10, Garmin). A description of sampling locations and coordinates is given in Additional file 1: Table S1. Mosquito collections were undertaken between June and July 2018.

### Mosquito sampling

**Fig. 2:**
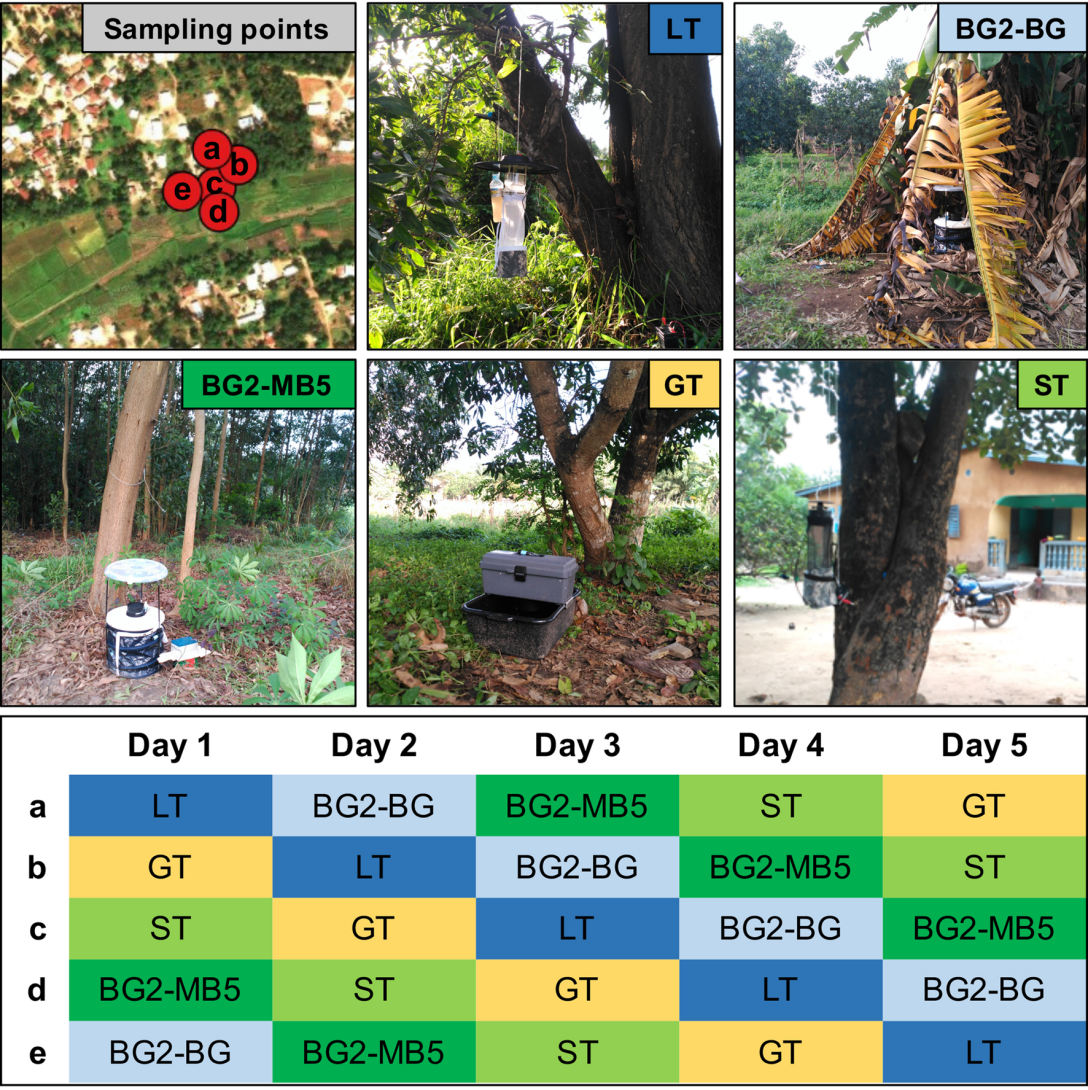
Example of distribution of traps in 5 sampling points in a 5 × 5 Latin Square design, in this case in Maferinyah Centre I, and the schedule for 5 days of collection. *Abbreviations*: LT, CDC light trap; BG2-BG, BG sentinel 2 with BG lure; BG2-MB5, BG sentinel 2 with MB5 lure; GT, Gravid trap; ST, Stealth trap

BG sentinel 2 traps (Biogents, Regensburg, Germany), CDC light traps (John W. Hock, Gainesville, Florida, USA), Reiter-Cummings gravid traps (BioQuip, Compton, California, USA) and Stealth traps (John W. Hock, Gainesville, Florida, USA) were used for mosquito collections. BG-lure (NH_3_, lactic acid and hexanoic acid) or BG-MB5 lure (NH_3_, lactic acid, tetradecanoic acid, 3-methyl-1-butanol and butan-1-amine) (Biogents, Regensburg, Germany) were used with BG2T (BG2-BG and BG2-MB5, respectively). To allow a direction comparison between the two lures, we did not include the use of carbon dioxide (CO_2_) with BG2T. ST is a novel trap that has not been widely tested to date and has eight ultraviolet LEDs in addition to an incandescent light which turn off automatically during the day. The portability of the ST (of a smaller weight and size than LT) is advantageous over significantly larger BG2T and GT. However, ST is more delicate than the LT since the operation mechanism is exposed, unlike in the LT, where it is protected inside the cover. The incandescent light of the LT was operational for 24 hours. CO_2_ was used as an attractant for LT and ST for the duration of the 24 h, directed into the vicinity of trap inlets using plastic containers. It was prepared by mixing 280 g of sugar and 5 g of yeast in 500 ml of water [25]. In each of the three sites, water collected locally from shallow sunlit ponds was used for the GR trap. A 5 × 5 Latin Square design was applied in each site (Fig. 2). The traps were placed in five sampling locations of one site at 19:00 h. Mosquitoes were collected every 12 hours and the traps were rotated to the next sampling point every 24 hours, so two collections (day and night) per trap per sampling point were obtained (Fig. 2). Since each site had 5 sampling points, each trap/site had a total of 10 collections. A total of 50 collections were obtained per site. Since this study was developed in 3 sites, a total of 150 collections were obtained (30 collections per trap) (Additional file 1: Table S2). Five HLCs were undertaken over 5 nights alongside mosquito trapping in Senguelen. Landing mosquitoes were collected outdoors from 20:00 to 02:00 h using manual aspirators in teams of 5 to 6 volunteers per night.

### Collection of environmental data

Temperature and relative humidity were recorded at each sampling point every 5 min using EL-USB-2 data loggers (Lascar Electronics, UK) and averaged over the 12-h period of each collection. Presence or absence of rain was recorded by field workers (Additional file 2: Figure S1).

### Identification of mosquitoes

Mosquitoes collected from traps and HLCs were morphologically identified using keys [26–28] and stored in RNAlater at − 80 °C. A subsample of 370 mosquitoes collected using traps was selected for molecular identification. At least one specimen of every morphologically identified species and unidentified specimens from each of the five traps and each of the three trapping locations were chosen for sequencing to confirm the identification. Genomic DNA was initially extracted from individual males morphologically identified as *Culex* (*Cx.*) using DNeasy-96 extraction kits (Qiagen, Manchester, UK) according to the manufacturer’s protocol with minor modifications. Genomic DNA extraction (as opposed to RNA extraction) was required as difficulties with ACE multiplex end-point PCR assays on cDNA used to determine species within the *Cx. pipiens* complex [29] has previously been observed. RNA extraction was undertaken on individual females morphologically identified as within the genera *Aedes*, *Anopheles* and *Eretmapodites* using RNeasy-96 extraction kits (Qiagen) according to the manufacturer’s protocol with minor modifications. RNA was reverse transcribed into complementary DNA (cDNA) using a High Capacity cDNA Reverse Transcription kit (Applied Biosystems, Warrington, UK). A final volume of 20 µl contained 10 µl RNA, 2 µl 10× RT buffer, 0.8 µl 25× dNTP (100 mM), 2 µl 10× random primers, 1 µl reverse transcriptase and 4.2 µl nuclease-free water. Cycling conditions were 25 °C for 10 min, 37 °C for 120 min and 85 °C for 5 min.

Different PCR assays were carried out depending on the genus. For discrimination of species of the *An. gambiae* complex, an end-point PCR to detect the *SINE200* insertion [30] and a multiplex PCR for amplification of an intergenic spacer (IGS) region [31] were used. Amplification and sequencing of regions of the cytochrome *c* oxidase subunit 1 (*cox*1) gene [32] and the internal transcribed spacer 2 (ITS2) region from the nuclear ribosomal DNA [33] was used for confirmation of *An. squamosus* and the rest of the *Anopheles* species collected, respectively. For identification of *Culex* species, amplification and sequencing of an alternative fragment of the *cox*1 gene [34] was used. Since this specific fragment did not provide enough variability to discriminate between *Cx. quinquefasciatus* and *Cx. p. pipiens*, an ACE multiplex end-point PCR assay [29] was used for discrimination. For identification of *Aedes* and *Eretmapodites*, in addition to confirmatory testing of *Cx.* cf. *sitiens* samples, amplification and sequencing of a further *cox*1 gene fragment [35] was undertaken. Primers and conditions of all PCR assays are described in Additional file 1: Table S3.

PCR assays were performed in a Bio-Rad T100 thermocycler and PCR products were visualised in precast Invitrogen 2% agarose E-gel cartridges (containing SYBR gold stain) in an E-Gel iBase power system (Invitrogen, Warrington, UK) using a 100 bp DNA ladder (NEB) for product size analysis. For barcoding, PCR products were submitted to Source BioScience (Source BioScience Plc, Nottingham, UK) for PCR reaction clean-up, followed by Sanger sequencing to generate both forward and reverse reads. Sequencing analysis was carried out in MEGA7 [36] as follows. Both chromatograms (forward and reverse traces) from each sample were manually checked, edited, and trimmed as required, followed by alignment with ClustalW and checking to produce consensus sequences. Consensus sequences were used to perform nucleotide BLAST (NCBI) database queries [37, 38]. Full consensus sequences were submitted to the GenBank database under the accession numbers MN545862–MN545865 (ITS2), and MN552290–MN552306 (*cox*1) (Additional file 1: Table S4). Confirmation of species was considered complete for sequences with an identity to a particular species given by BLAST of greater or equal to 98%, and where no other species also gave identities at this level.

### Data analysis

Functions “filter”, “select”, “group_by”, “n” and “summarise” from package *dplyr* [39] were used in RStudio [40] for data handling. A Generalised Linear Mixed Model (GLMM) with the Negative Binomial distribution was applied to the data with the function “glmer.nb” from package *lme4* [41] in RStudio to compare the effect of the traps, sites and collection times on the abundance of mosquitoes. Function “glht” from package *multcomp* [42] was used for multiple comparisons between the levels of each fixed effect. *Trap*, *Time* and *Site* were included as fixed effects. *Sampling point* was included as a random factor. *Temperature* and *Humidity* were included as covariates; with *Rainfall* included as a binary factor. ANOVA was used to compare model fit by stepwise deletion of non-significant variables, using the Aikaike information criterion (AIC) as an indicator of a better model fit. Simpson’s diversity index per *Trap*, *Site* and *Time* was calculated to compare the species diversity. Simpson’s diversity index indicates a high diversity when it is close to 0 and low diversity when it is close to 1.

## Results

### Comparison of five adult mosquito traps

A total of 10,610 mosquitoes were trapped by the five adult mosquito traps across the 30 collection intervals (15 days and 15 nights) of the study. In terms of abundance, the ST captured the highest percentage of the total number of mosquitoes collected (67%), followed by the LT (24%), the BG2-MB5 lure (4%), the GT (3%) and the BG2-BG lure (2%) (Table 1). The diversity of species was measured using the Simpson’s diversity index. Results showed that the BG2-BG captured the most diverse range of mosquito species (Simpson’s diversity index of 0.157), followed by the GT (0.241), BG2-MB5 (0.24), LT (0.415) and ST (0.484) (Table 1).

**Table 1:** Diversity and relative abundance of mosquitoes by trap

Specimen and condition	BG sentinel BG lure	BG sentinel MB5 lure	CDC light trap	Gravid trap	Stealth trap
*Aedes*					
Blood-fed F	0	5	18	0	4
Gravid F	20	20	161	18	25
Unfed F	46	28	3	17	374
Unknown F status	0	0	0	0	6
Male	12	8	72	1	63
Unknown sex	1	0	2	0	1
Subtotal (%)	79 (36.92)	61 (13.29)	256 (10.05)	36 (12.20)	473 (6.67)
*Anopheles*					
Blood-fed F	1	7	3	0	4
Gravid F	0	17	1	4	1
Unfed F	47	78	81	6	198
Unknown F status	0	0	2	0	5
Male	1	7	7	8	45
Unknown sex	2	1	0	0	2
Subtotal (%)	51 (23.83)	110 (23.97)	94 (3.69)	18 (6.10)	255 (3.59)
*Culex*					
Blood-fed F	1	2	13	5	21
Gravid F	7	34	172	105	327
Unfed F	56	184	1089	77	3165
Unknown F status	0	0	23	0	187
Male	18	63	888	54	2586
Unknown sex	0	0	2	0	9
Subtotal (%)	82 (38.32)	283 (61.66)	2187 (85.9)	241 (81.69)	6295 (88.71)
Unidentified Culicines					
Gravid F	0	0	1	0	1
Unfed F	1	0	2	0	17
Unknown F status	0	0	0	0	10
Male	0	3	4	0	21
Unknown sex	0	0	0	0	1
Subtotal (%)	1 (0.47)	3 (0.65)	7 (0.27)	0	50 (0.70)
*Eretmapodites*					
Gravid F	1 (0.47)	0	0	0	0
*Mansonia*					
Unfed F	0	2 (0.44)	0	0	0
*Uranotaenia*					
Unfed F	0	0	2 (0.08)	0	1 (0.02)
Unidentified specimens					
Unknown sex	0	0	0	0	22 (0.31)
No. of mosquitoes	214	459	2546	295	7096
No. of species	12	14	14	13	19
Simpsonʼs diversity index	0.157	0.24	0.415	0.241	0.484

*Notes*: The number of mosquitoes from each genus is split into sex (male, female, unknown) and female (F) status (blood-fed, gravid, unfed, unknown). An unknown sex or status is caused by significant damage of the specimen. The subtotals show the proportion of each genus in relation with the total number of mosquitoes collected within each trap

The majority of the mosquitoes collected across this study belonged to the genera *Anopheles*, *Aedes* and *Culex*. However, the ST and LT captured one and two *Uranotaenia* mosquitoes respectively, the BG2-MB5 captured two *Mansonia* and the BG2-BG captured one *Eretmapodites* (Table 1). Regarding the sex of collected mosquitoes, 38% of the specimens captured by the LT and ST were males, whereas for the other traps, males were less than 22%. GT caught the highest proportion of gravid females, whereas unfed females represented the highest proportion of the catch in other traps. Blood-fed females made up the smallest group, with the BG2-MB5 trapping the highest relative proportion. The total numbers of blood-fed females were too low for comparative blood-meal analysis (Table 1).

**Fig. 3:**
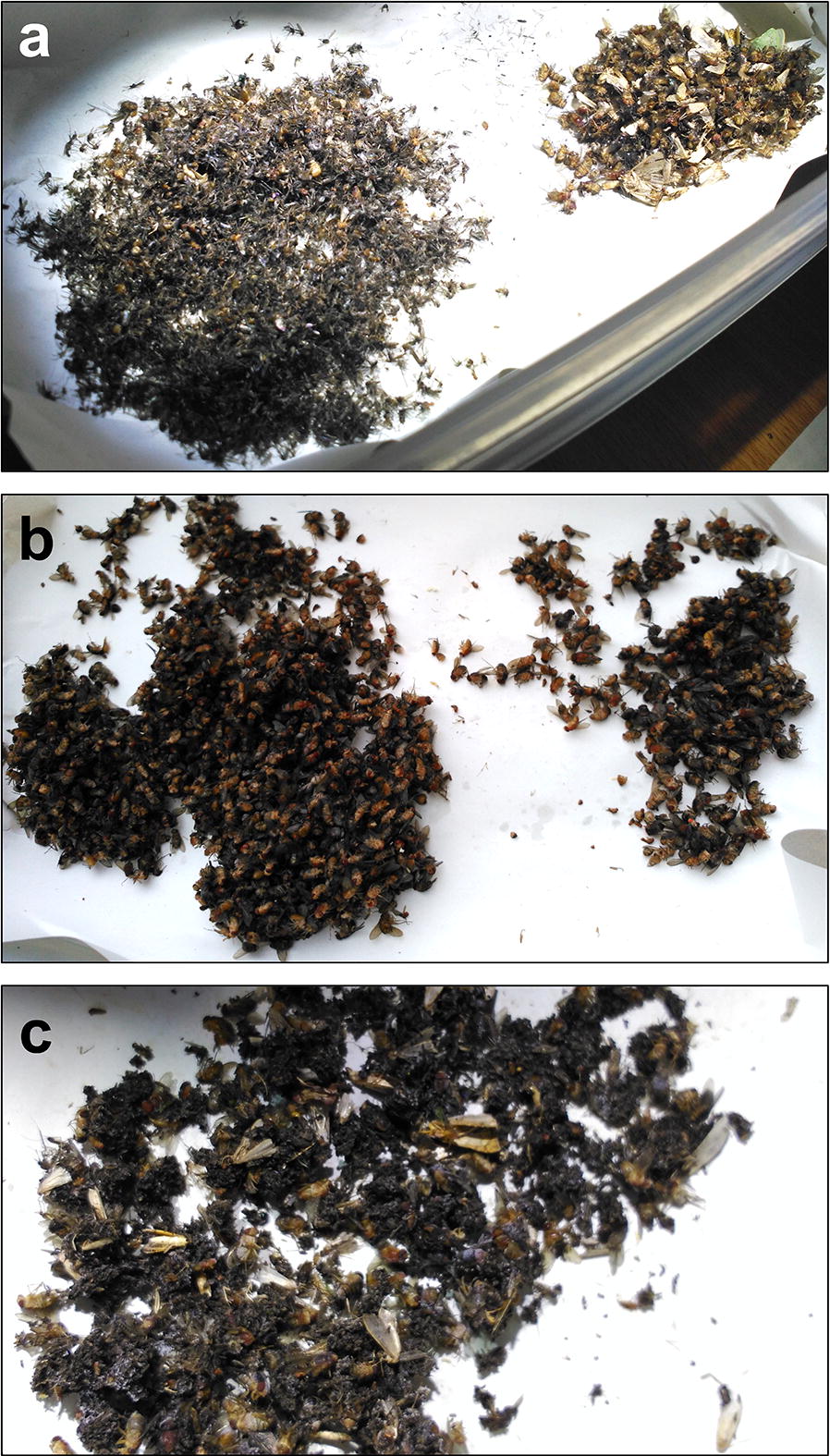
Examples of 12-hour collections of the ST. **a** The largest collection of the study, showing a bigger group (left) containing a majority of mosquitoes and a smaller group (right) with unidentified Diptera and other insects already sorted. In this collection and others, some mosquitoes were being eaten by ants. **b** Collection with the largest number of unidentified Diptera, which mask the presence of mosquitoes, also abundant. **c** Collection with the largest number of damaged mosquitoes, which were wet and stuck to each other and to small unidentified Diptera

‘Damage state’ of the specimens was also annotated and assessed. No specimens were damaged by the gravid trap, less than 10% of the specimens were damaged in both BG2T and 10% of specimens were damaged in the LT (data not shown). However, the ST resulted in the highest proportion of damaged mosquitoes at approximately 20%, of which nearly one quarter could not be morphologically identified (Table 1). Although the ST captured the largest number of mosquitoes, this trap also collected a large number of non-target Diptera and ants, making sorting of the specimens time-consuming (Fig. 3).

### Generalised linear mixed model for mosquito abundance

A negative binomial GLMM was used to determine statistical differences between the abundance of mosquitoes captured by each trap. The results indicated that the following parameters influenced the number of mosquitoes collected: *Site* (Maferinyah Centre I, Senguelen and Fandie), *Time Period* (evening and morning), *Trap* (BG2-BG, BG2-MB5, GT, LT, ST) and *Sampling Point* (random factor). *Rainfall*, *Temperature* and *Humidity* did not significantly influence the data, so they were removed from the model. The final, best-fit model was: *Abundance~Site+(1|Point)+Time+Trap*. According to this model, there were no significant differences between the abundance of mosquitoes captured by the GT, the BG2-MB5 and the BG2-BG (Additional file 1: Table S5). However, there were significant differences between the abundance of mosquitoes captured by GT and LT (Tukey, *Z* = − 3.41, *df* = 145, *P* = 0.006), between LT and BG2-MB5 (Tukey, *Z* = 3.64, *df* = 145, *P* = 0.003) and between LT and BG2-BG (Tukey, *Z* = 4.16, *df* = 145, *P* < 0.001). Also, significant differences were found between the abundance of mosquitoes captured by the ST and all the rest of the traps: ST and BG2-MB5 (Tukey, *Z* = 6.94, *df* = 145, *P* < 0.001), ST and BG2-BG (Tukey, Z = 7.38, *df* = 145, *P* < 0.001), ST and GT (Tukey, *Z* = 6.53, *df* = 145, *P* < 0.001) and ST and LT (Tukey, *Z* = 3.46, *df* = 145, *P* = 0.005) (Additional file 1: Table S4). Regarding sites and collection intervals, more mosquitoes were captured in Senguelen than in Maferinyah Centre I (Tukey, *Z* = 5.03, *df* = 87, *P* = 0.0004) and Fandie (Tukey, *Z* = 3.78, *df* = 87, *P* = 0.0005) and significantly more mosquitoes were captured during the night than during the day (Tukey, *Z* = − 10.52, *df* = 58, *P* < 0.0001).

The above model was used to assess the effectiveness of the different traps at capturing *Aedes*, *Anopheles* and *Culex* mosquitoes in general, and *An. gambiae* (*s.l*.) and *Ae. aegypti* species in particular, since they are the main vectors of disease (Table 2). The results showed that the ST was the best trap at capturing *Aedes* mosquitoes, although it only showed to be significantly better than the GT (Tukey, *Z* = 3.47, *df* = 145, *P* = 0.005). Both BG2T were significantly better overall at capturing *Ae. aegypti* mosquitoes: BG2-BG *vs* LT (Tukey, *Z* = − 2.74, *df* = 145, *P* = 0.045); BG2-BG *vs* ST (Tukey, *Z* = − 3.32, *df* = 145, *P* = 0.008); BG2-MB5 *vs* LT (Tukey, *Z* = − 3.57, *df* = 145, *P* = 0.003); BG2-MB5 *vs* GT (Tukey, *Z* = − 2.92, *df* = 145, *P* = 0.027); BG2-MB5 *vs* ST (Tukey, *Z* = − 3.92, *df* = 145, *P* < 0.001). The ST collected the greatest number of *Anopheles* spp. and *An. gambiae* (*s.l*.) in particular, although no significant differences were seen when compared with the other traps. The ST captured significantly more *Culex* mosquitoes than both BG2T and GT (Tukey, *df* = 145, *P* < 0.001) and that LT (Tukey, *Z* = 3.54, *df* = 145, *P* = 0.003). Finally, the LT captured significantly more *Culex* than both BG2T (Tukey, *df* = 145, *P* < 0.001) and GT (Tukey, *Z* = − 2.74, *df* = 145, *P* = 0.048).

**Table 2:** Statistical differences between the abundance of *Anopheles* spp., *Aedes* spp. and *Culex* spp. and *An. gambiae* (*s.l*.) and *Ae. aegypti* mosquitoes captured by the five traps

Mosquito genus/complex/species	BG sentinel BG lure	BG sentinel MB5 lure	CDC light trap	Gravid trap	Stealth trap
*An. gambiae* (*s.l*.)	1.63^a^ (0.61–2.66)	3.60^a^ (2.00–5.20)	2.67^a^ (1.10–4.23)	0.53^a^ (0.04–1.03)	7.93^a^ (5.26–10.61)
*Ae. aegypti*	1.00^a^ (0.38–1.62)	1.37^ac^ (0.82–1.91)	0.23^b^ (-0.19–0.65)	0.37^abd^ (0–0.73)	0.10^b^ (-0.25–0.45)
*Aedes* spp.	2.37^ab^ (1.57–3.16)	2.13^ab^ (1.65–2.62)	8.53^ab^ (5.92–11.14)	1.13^b^ (0.73–1.54)	15.73^a^ (10.77–20.70)
*Anopheles* spp.	1.73^a^ (0.68–2.79)	3.60^a^ (1.99–5.21)	3.20^a^ (1.70–4.70)	0.67^a^ (0.15–1.19)	8.5^a^ (5.85–11.15)
*Culex* spp.	2.83^a^ (1.57–4.10)	9.43^ade^ (6.61–12.26)	72.93^bd^ (68.15–77.72)	8.07^de^ (6.84–9.30)	209.87^c^ (200.60–219.14)

*Notes*: Mean number (and 95% confidence interval) of mosquitoes captured per collection interval per trap are shown. The values in each row are significantly different from each other if they do not share the same superscript letter

### Comparison of *An. gambiae* complex species collected using HLCs and adult mosquito traps

**Fig. 4:**
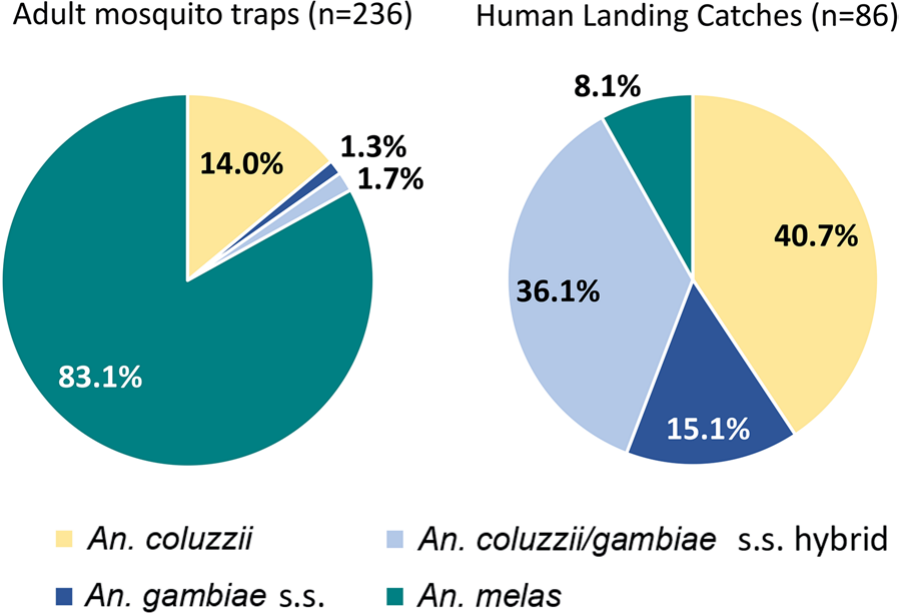
Comparison of species from the *An. gambiae* complex captured by adult mosquito traps (left) and HLCs (right)

A total of 1940 *An. gambiae* (*s.l*.) females were collected from Senguelen across 5 nights. Randomly selected subsamples of 86 and 236 specimens of the *An. gambiae* (*s.l*.) mosquitoes collected from Senguelen using HLCs and adult mosquito traps respectively, were selected for molecular identification and comparison of species composition (Fig. 4). Results showed that *An. melas* was the predominant species (85%) caught by adult mosquito traps, whereas it was collected at the least frequency (10%) using HLCs. *Anopheles coluzzii* and *An. gambiae* × *An. coluzzii* hybrids were the most abundant species collected using HLCs (40% and 35% respectively), whereas these were 12% and 2% of the collections, respectively, using adult traps. *Anopheles gambiae* represented 15% of the individuals collected using HLCs whereas this species was only 1% of the individuals collected using adult traps.

### Species composition in the Maferinyah subprefecture

Senguelen was the site with the highest number of mosquitoes (5784) followed by Fandie (4094) and Maferinyah Centre I (732) (Table 3). The diversity of the species from the day collection (07:00 to 19:00 h) was similar to the night collection (19:00 to 07:00 h) in Senguelen and Maferinyah Centre I, presenting a Simpson’s diversity index of around 0.2 and 0.3 respectively. However, Fandie showed a high diversity in the day collection (0.142) and a low diversity in the night collection (0.48) (Table 3). A total of 25 species were found across the three sites (using a combination of morphological and/or molecular identification), belonging to the genera *Aedes*, *Anopheles*, *Culex*, *Eretmapodites*, *Mansonia* and *Uranotaenia*. One *Toxorhynchites brevipalpis* was also captured during a morning collection in Fandie by the BG2-BG. However, the power failed to one of the traps during this round, and therefore the collection could not be included in the analysis.

**Table 3:** Diversity and relative abundance of mosquitoes per site and collection interval

Site	Collection Period	No. of mosquitoes (%)	No. of species	Simpsonʼs diversity index
Fandie	Night	4031 (38.0)	14	0.480
	Day	63 (0.6)	9	0.142
Maferinyah Centre I	Night	690 (6.5)	17	0.346
	Day	42 (0.4)	5	0.383
Senguelen	Night	5256 (49.5)	19	0.274
	Day	528 (5)	10	0.220
Total		10,610	25	

*Notes*: Percentages (%) show the proportion of mosquitoes collected in each site (and collection interval) in relation with the total number of mosquitoes. Simpson’s diversity index indicates a high diversity when it is close to 0 and low diversity when it is close to 1

A subsample of 370 specimens were selected for molecular identification. This subsample included 249 *Anopheles*, 24 *Aedes*, 96 *Culex* and 1 *Eretmapodites* individual. These numbers represented 47.2%, 2.7%, 1.1% and 100%, respectively, of the total number of collected mosquitoes within each genus (Additional file 1: Table S6A). The 370 specimens selected for molecular identification were chosen in order to confirm the species identity of mosquitoes collected using all traps across the three sites, representing 1.4%, 8.5% and 4.4% of the total collections from Fandie, Maferinyah Centre I and Senguelen, respectively (Additional file 1: Table S6B).

In total, 20 species were confirmed by Sanger sequencing (Additional file 1: Table S4). *An. coustani* was confirmed by sequencing a fragment of the ITS2 region. A combination of ITS2 fragment sequencing [33] and species-specific end-point PCRs [30, 31] allowed the identification of the following species of the *An. gambiae* complex: *An. gambiae*, *An. coluzzii* and *An. melas*. *Anopheles squamosus* was confirmed by sequencing a fragment of the *COI* gene [32]. Sequencing of a different fragment of the *cox*1 gene [34] confirmed the presence of *Lt. tigripes, Cx. watti* and individuals from the *Cx. pipiens* complex. A combination of the *cox*1 gene fragment sequencing and the ACE multiplex PCR [29] confirmed the presence of *Cx. pipiens*, *Cx. quinquefasciatus* and hybrids in Guinea. Sequences with 94.88% identity to the species *Cx. watti* were also generated, but this would more likely be indicative of a closely related species with no sequences available on GenBank currently. Top BLAST results from some *Culex* individuals resulted in most significant alignments with *Cx. sitiens* sequences, generating maximum identities ranging from 97.19% to 97.64% with this fragment of the *cox*1 gene [34]. Further confirmation attempts of these individuals, utilising one of the alternative *cox*1 fragments [35] as geographically closer *Cx. sitiens* GenBank sequences (from Kenya) were available for comparison for this fragment, resulted in maximum identities of 97.57%. Although these identities are just below the 98% threshold, it is likely this species is *Cx. sitiens*, but that the sequences from Guinea exhibit genetic variation to those for this species currently available on GenBank, or, that this is a very closely related species. To avoid the possibility of inaccurate confirmation, individuals from this species are referred to as *Cx.* cf*. sitiens*. Sequencing of the alternative *cox*1 fragment [35] confirmed the following *Aedes* species: *Ae. aegypti*, *Ae. vittatus*, *Ae. fowleri*, *Ae. cumminsi*, *Ae. argenteopunctatus* and a species within the *Ae. simpsoni* complex. Top BLAST results for *Aedes* individuals that resulted in *Ae. luteocephalus* and *Ae. denderensis* presented a maximum identity of 91.19 and 92.14%, respectively, suggesting these individuals were closely related species which have no sequences currently available in GenBank. The analysis of the same *cox*1 sequence [35] also confirmed the presence of *Er. intermedius*.

## Discussion

To our knowledge, this study provides the first entomological survey in Guinea that compares the mosquito species abundance and diversity using a range of different adult mosquito traps. Other studies in West Africa have utilised some of these traps individually, such as LT in Guinea [22] and Sierra Leone [43], and GT in Ghana [9]. This is also the first study that compares the performance of a ST with other mosquito traps to catch mosquitoes in a field setting. The results presented in our study show significant differences in the abundance of mosquitoes captured by the ST and the rest of the traps. The ST captured the greatest number of mosquitoes, followed by the LT, BG2T with MB5 lure (BG2-MB5), GT and BG2T with BG lure (BG2-BG). Therefore, the use of LT, and particularly ST, would be recommended for studies that are aiming to obtain large numbers of particular mosquito species. The fact that ST captured significantly more mosquitoes than LT (*P* = 0.00492) is surprising considering that their performance is similar: when the light attracts the mosquitoes, they get trapped after passing through a fan. The addition of a UV light, a smaller size and black and camouflage fabric are the only features that make the ST different to the LT. The ST can be used in four different ways by combining two types of light and the presence or absence of CO_2_. For this study, both lights and CO_2_ were used, so further studies should compare the efficacy of the ST when performing with the other combinations. Since the ST, followed by the LT, captured the highest proportion of male mosquitoes, they could be utilised in studies looking at male behaviour. In general across the traps, sites and collection intervals, all the study collections presented a greater number of females than males. However, interestingly this composition was reverted in two collections, and a greater number of males was captured in sampling points C and E in Fandie. The fact that these two sampling points may have been located next to a swarm could be a potential explanation [44].

Previous studies suggest that the LT are optimal for catching *Anopheles* [45]; however, the main genus captured by the LT was *Culex*. In contrast, the ST was the best at capturing the largest number of *Anopheles* mosquitoes in general and *An. gambiae* (*s.l*.) in particular. According to Costa-Neta et al. [46], the higher the intensity of the light source, the higher the number of *Anopheles* captured. This may be one reason why the ST captured the greatest number of *Anopheles* (Tables 1 and 2). Previous studies also suggest that the GT are good at catching *Culex* [47], and this was indeed the main genus captured by this trap. However, the ST collected significantly more *Culex* mosquitoes than the GT (Tables 1 and 2). As expected, the GT also captured the highest proportion of gravid females. Additionally, all of the specimens were un-damaged, since the design of the trap allows the collection of specimens without passing through a fan, so its use could be beneficial to capture mosquitoes with the objective of establishing a colony or screening for arbovirus transmission.

Due to the small sample size, no conclusions can be made regarding the best collection method for *Eretmapodites*, *Mansonia* and *Uranotaenia* mosquitoes. Although the ST showed the best performance in terms of abundance of mosquitoes captured, this trap also caused significant damage to specimens, making morphological identification time-consuming and inaccurate. One reason for this damage could be the high density of collected specimens (Fig. 3a), which remained in the trap for up to 12 hours during trapping intervals, depending on trap entry time. In addition to this, the presence of ants and big Diptera could have also contributed to this damage (Fig. 3a, b). Another reason could be the low protection that this trap confers to the collected specimens from rainfall, due to the small surface area of the cover/rain shield, resulting in wet and clumping specimens (Fig. 3c). Therefore, the performance of the ST could potentially be improved by using it for shorter periods of time or by swapping collection bags more often, to reduce the high densities of mosquitoes within the same collection bag. Also, by choosing locations offering greater protection from rainfall, which could help reduce damage to the specimens.

The BG lure is designed to attract mainly *Aedes* whereas the MB5 lure was specifically designed for *Anopheles* [48, 49]. Although BG2T with BG lure have been used in Burkina Faso [50], to our knowledge no traps have been used in West Africa with the MB5 lure so far, so both lures were tested in the two BG2T in this study. Previous studies suggest that the BG2T in general are effective for catching *Aedes* mosquitoes [51], and that the addition of the BG lure improves this [51]. In this study, no significant differences were seen in the number of *Aedes* mosquitoes (at genus level) captured by the five different traps, although the high proportion of *Aedes* specimens captured by the BG2-BG (Table 1), in comparison with the rest of the traps, suggests the composition of the BG lure is good at attracting this genus in particular. This finding also supports previous studies which have also shown the good performance of this trap-lure combination at capturing *Ae. aegypti* mosquitoes in Brazil [52]. Additionally, both BG2T presented the best performance at capturing *Ae. aegypti* mosquitoes in comparison with the rest of the traps, with no differences between the two lures (Table 2), suggesting two possibilities: first, it is the design of the trap and not the lure that works so well at capturing *Ae. aegypti* mosquitoes. Secondly, the addition of the lure improves the attraction of *Ae. aegypti* mosquitoes but no difference is present between the BG and the MB5 lures at attracting this species. Both BG2T demonstrated effective performance at capturing *Anopheles* mosquitoes (as reported by Pombi et al. [50]). The MB5 lure was designed for attracting *Anopheles* mosquitoes [49], and indeed it was demonstrated to be better than the BG2-BG at capturing *Anopheles* mosquitoes and in particular *An. gambiae* (*s.l*.). However, no significant differences were detected between both (Table 2), indicating that the MB5 lure needs further improvement in order to obtain more effective collections of this genus. Furthermore, these results are also only comparing lures without the addition of CO_2_, so further studies are needed to determine the impact of CO_2_ on the efficacy of BG2T. The use of lures in BG2T also introduces an inherit bias given the lures are designed to specifically attract individual species. Although the number of *Anopheles* (and *An. gambiae* (*s.l*.)) captured by both BG2T was lower than the number captured by the ST, no significant differences were observed between the ST and the two BG2T (Table 2). Therefore, BG2T could be used for studies specifically looking at *Anopheles*. According to the results of this study, an increased number of trapping intervals would be recommended for BG2T use to increase the number of *Anopheles* mosquitoes captured. However, we are aware that the BG2T are commonly used with CO_2_, and that its replacement with two lures may be the reason why these traps did not capture as many mosquitos as LT and ST (used with CO_2_) in this study. Thus, further assessment of the BG2T used with different baits (including CO_2_) would be necessary to determine the optimal performance of this trap in this area.

Diversity takes into account richness (number of different species) and evenness (comparison of population size of each species). Although the number of species captured by the LT and ST was higher than the other traps (high richness), the difference in the number of specimens from each species was higher than the other traps (low evenness). Therefore, the diversity of the mosquito populations captured by LT and ST was the least diverse. The BG2-BG presented the most diverse collection of mosquitoes, followed by the GT and the BG2-MB5. Although our data suggest these traps could be used in studies looking for maximum species diversity, it could also be observed that the BG2T failed to collect large numbers of the most abundant species, although this could be due to the lack of CO_2_. In contrast, LT and ST would be recommended for studies requiring a large number of mosquitoes of a particular species, with exception of some species (see Table 4).

**Table 4:** Mosquito species captured per trap, site and collection period

Traps, sites and collection periods	Species
Species captured by trap	
BG sentinel 2 BG lure	*Ae. aegypti*, *Ae. argenteopunctatus*, *Ae. cumminsi*, *Ae.* cf*. luteocephalus*, *Ae. simpsoni* (*s.l.*)*, An. coluzzii*, *An. gambiae* (*s.s*.), *An. melas*, *Cx. pipiens*, *Cx. quinquefasciatus*, *Cx.* cf*. sitiens*, *Er. intermedius*
BG sentinel 2 MB5 lure	*Ae. aegypti*, *Ae. argenteopunctatus*, *Ae. simpsoni* (*s.l*.), *Ae. cumminsi*, *Ae.* cf*. luteocephalus*, *An. coluzzii*, *An gambiae* (*s.s*.), *An. gambiae/An. coluzzii* hybrid, *An. melas*, *Cx. pipiens*, *Cx. quinquefasciatus*, *Cx.* cf*. sitiens*, *Cx. watti*, *Mansonia* spp.
CDC light trap	*Ae. aegypti*, *Ae. argenteopunctatus*, *Ae. simpsoni* (*s.l*.), *Ae. cumminsi*, *An. coluzzii*, *An. coustani* (*s.l*.), *An. melas*, *An. squamosus*, *Cx. pipiens*, *Cx. quinquefasciatus*, *Cx.* cf*. sitiens*, *Lt. tigripes*, *Cx. watti*, *Uranotaenia* spp.
Gravid trap	*Ae. aegypti*, *Ae. cumminsi*, *Ae.* cf*. denderensis*, *Ae.* cf*. luteocephalus*, *Ae. simpsoni* (*s.l*.), *An. coluzzii*, *An. melas*, *An. squamosus*, *Cx. pipiens*, *Cx. quinquefasciatus*, *Cx.* cf*. sitiens*, *Cx.* cf*. watti*, *Cx. watti*
Stealth trap	*Ae. aegypti*, *Ae. argenteopunctatus*, *Ae. cumminsi*, *Ae. fowleri*, *Ae. vittatus*, *An. coluzzii*, *An. coustani* (*s.l*.), *An. gambiae* (*s.s*.), *An. gambiae/An. coluzzii* hybrid, *An. melas*, *An. obscurus*, *An. squamosus*, *Cx. pipiens*, *Cx. pipiens/Cx. quinquefasciatus* hybrid, *Cx. quinquefasciatus*, *Cx.* cf*. sitiens*, *Lt. tigripes*, *Cx. watti*, *Uranotaenia* spp.
Species captured by site	
Fandie	*Ae. aegypti*, *Ae. argenteopunctatus*, *Ae. cumminsi*, *Ae.* cf*. denderensis*, *Ae.* cf*. luteocephalus*, *Ae. simpsoni* (*s.l*.), *An. coluzzii*, *An. coustani* (*s.l*.), *An. gambiae* (*s.s*.), *An. gambiae/An. coluzzii* hybrid, *An. melas*, *Cx. pipiens*, *Cx. quinquefasciatus*, *Cx.* cf*. sitiens*, *Lt. tigripes*, *Uranotaenia* spp.
Maferinyah Centre I	*Ae. aegypti*, *Ae. cumminsi*, *Ae. fowleri*, *Ae.* cf*. luteocephalus*, *Ae. simpsoni* (*s.l*.), *Ae. vittatus*, *An. coluzzii*, *An. coustani* (*s.l*.), *An. gambiae* (*s.s*.), *An. melas*, *An. squamosus*, *Cx. pipiens*, *Cx. pipiens/Cx. quinquefasciatus* hybrid, *Cx. quinquefasciatus*, *Cx.* cf*. sitiens*, *Cx.* cf*. watti*, *Cx. watti*
Senguelen	*Ae. aegypti*, *Ae. argenteopunctatus*, *Ae. cumminsi*, *Ae.* cf*. luteocephalus*, *Ae. simpsoni* (*s.l*.), *An. coluzzii*, *An. coustani* (*s.l*.), *An. gambiae* (*s.s*.), *An. gambiae/An. coluzzii* hybrid, *An. melas*, *An. obscurus*, *An. squamosus*, *Cx. pipiens*, *Cx. quinquefasciatus*, *Cx.* cf*. sitiens*, *Lt. tigripes*, *Cx. watti*, *Er. intermedius*, *Mansonia* spp.
Species captured by collection period	
Day	*Ae. aegypti*, *Ae. cumminsi*, *Ae.* cf*. denderensis*, *Ae.* cf*. luteocephalus*, *Ae. simpsoni* (*s.l*.), *An coluzzii*, *An. melas*, *Cx. pipiens*, *Cx. quinquefasciatus*, *Cx.* cf*. sitiens*, *Lt. tigripes*, *Cx. watti*
Night	*Ae. aegypti*, *Ae. argenteopunctatus*, *Ae. simpsoni* (*s.l*.), *Ae. cumminsi*, *Ae. fowleri*, *Ae.* cf. *luteocephalus*, *Ae. vittatus*, *An. coluzzii*, *An. coustani* (*s.l*.), *An. gambiae* (*s.s*.), *An gambiae/An. coluzzii* hybrid, *An. melas*, *An. obscurus*, *An. squamosus*, *Cx. pipiens*, *Cx. pipiens/Cx. quinquefasciatus* hybrid, *Cx. quinquefasciatus*, *Cx.* cf*. sitiens*, *Cx.* cf*. watti*, *Lt. tigripes*, *Cx. watti*, *Er. intermedius*, *Mansonia* spp., *Uranotaenia* spp.

Human landing catches are the gold standard method for measuring exposure of humans to mosquito bites [53]. However, this method is labour-intensive and faces ethical considerations [54], as operators are potentially exposed to pathogens during collections. Since adult mosquito traps are an affordable and easy to use alternative which provides reliable entomological data about malaria transmission [55], we compared both methods specifically targeting the major malaria vectors in the *An. gambiae* complex. Human landing catches captured predominantly *An. coluzzii*, *An. gambiae* and hybrids, but they only captured a small percentage of *An. melas*. Alternatively, more than three quarters of the trap collections were *An. melas* and only a small percentage was *An. coluzzii*, followed by a smaller proportion of *An. gambiae* and hybrids. *Anopheles gambiae* and *An. coluzzii* are highly anthropophilic, whereas *An. melas* is considered opportunistic, feeding on humans when available and on other mammals otherwise [56]. Although different cues such as lights and lures that mimic human odours are used in mosquito traps to try to attract host-seeking females, as expected, HLCs are more effective at attracting anthropophilic *Anopheles* species. Therefore, this method would still be recommended for targeting species with this behaviour. These results also suggest that an improvement in lures or trap design is needed to better mimic human cues and increase the number of anthropophilic species captured. Some studies have tried this in the past by modifying BG sentinel traps to increase the captures of *An. darlingi* [57] and *An. arabiensis* [58] mosquitoes and use them as an alternative for HLCs. However, others have also shown that HLCs are still more effective at capturing *Anopheles* species in comparison with adult traps, whose main collections comprise culicines [59], as seen in the present study. Since in our study both methods (HLCs and mosquito traps) were undertaken outdoors, no conclusions can be made about which method would work more effectively for targeting different feeding and resting behaviours.

Senguelen, a rural site, presented the highest relative abundance of mosquitoes, whereas Maferinyah Centre I, a semi-urban site, presented the lowest relative abundance. In terms of mosquito species diversity, the former was also more diverse than the latter. The fact that the rural site was surrounded by dense vegetation and breeding sites, as opposed to the semi-urban environment, could explain these differences. Both Senguelen and Maferinyah Centre I presented similar diversities between day and night collections. However, Fandie presented the highest diversity during the day and the lowest diversity during the night, likely due to the most diverse range of day-biting species present in this semi-rural area. As expected, the abundance of mosquitoes captured during the night was significantly higher than the day collection, since some of the most abundant mosquitoes of the collection, such as *Cx. quinquefasciatus*, are night biters. The highly abundant *Cx.* cf*. sitiens* were also mostly collected at night, indicating similar behaviour to C*x. sitiens* which are known night biters [60]. Some day-biting mosquitoes, like *Ae. aegypti*, may have been found in the night collection, as well as some night biters, like *An. gambiae* (*s.l*.), may have been found in the day collection, likely due to the inclusion of dawn and dusk in the night and day collections respectively.

Traditionally, identification of mosquitoes has been carried out using morphology. Although morphological identification is faster and more economical for large numbers of specimens, inaccuracies can result from specimens that do not present obvious and exclusive features. We used molecular identification in this study to confirm the identity of at least one specimen of every morphologically identified species from each of the five traps and each of the three trapping locations to provide greater certainty on our morphological identification in addition to unidentified specimens.

**Fig. 5:**
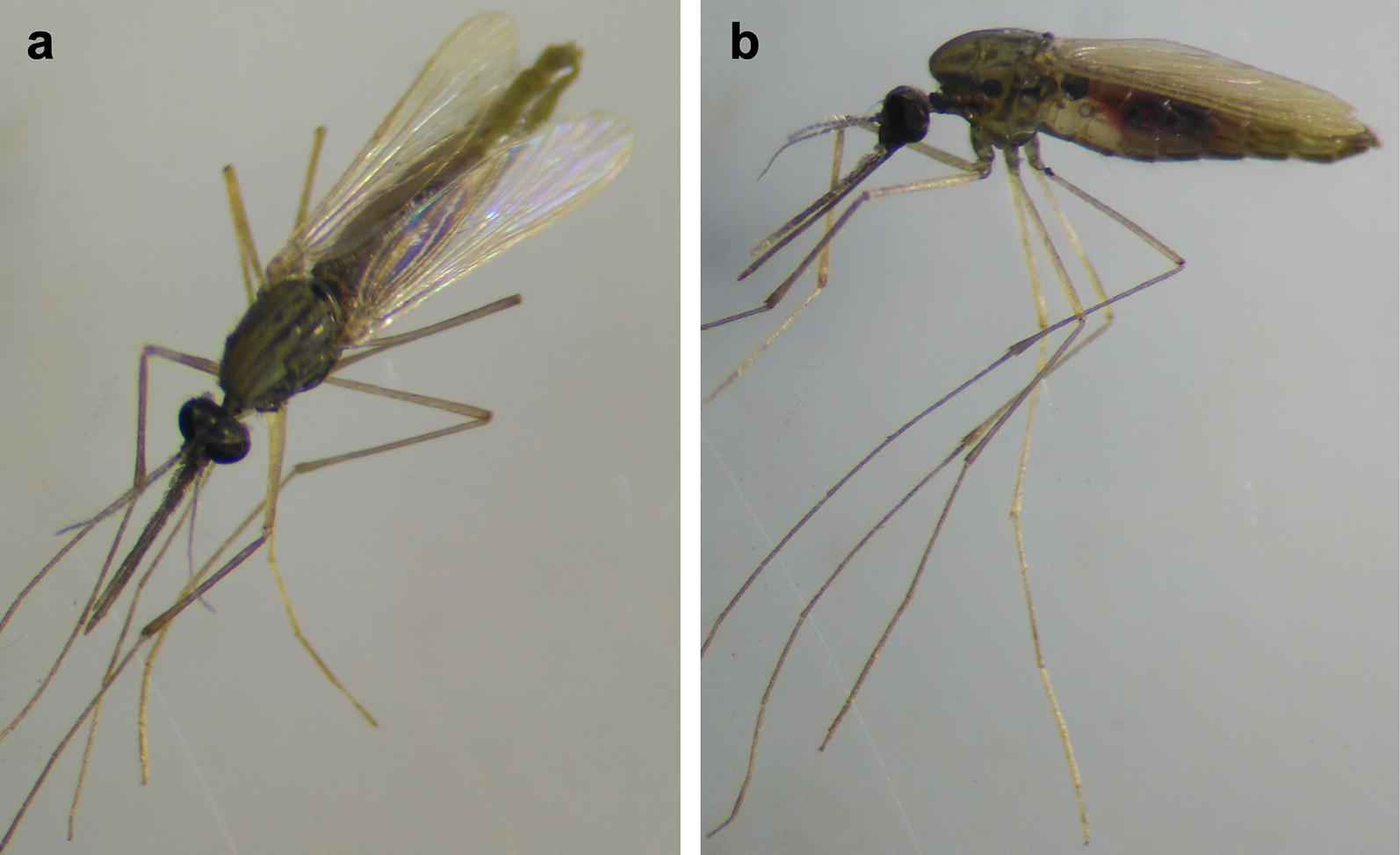
Morphologically unidentified golden-colour *Anopheles* female mosquito. Specimen morphologically identified as an *Anopheles* spp. and confirmed using PCR and sequencing as *An. coluzzii* from top (**a**) and lateral (**b**) view

As an example, one of the female mosquitoes collected using HLCs presented long palps, typical for the genus *Anopheles*, but it was white in colour and did not present the common wing and leg patterns of many species of the *Anopheles* genus (Fig. 5). This individual female could not be identified by experienced entomologists using *Anopheles* keys so DNA was extracted from this individual and PCR with Sanger sequencing revealed this species to be *An. coluzzii.* Random mutagenesis could be a potential explanation for this phenotype. Since molecular tools can complement and improve morphological identification of mosquitoes, it would be recommended to combine both for further entomological investigations.

Among the species whose presence was confirmed in Guinea using molecular methods, we identified important vectors of disease such as *An. gambiae* and *Ae. aegypti*. This suggests the potential for transmission of malaria, lymphatic filariasis and also several arboviruses of medical importance in this area of Guinea. Although they were found in Guinea, no evidence of pathogens transmitted by *Cx. watti* and *Lt. tigripes* was found from literature searches. The specimen from the genus *Eretmapodites* collected during this study was confirmed to be *Er. intermedius*. However, only *Er. silvestris*, *Er. inomatus* and *Er. quinquevittatus* have been found to be positive for Spondweni virus, Zika virus and Rift Valley fever virus, respectively [61]. *Mansonia uniformis* and *Uranotaenia mashonaensis* (both previously reported in Guinea) have been confirmed to be vectors of disease, but since no confirmation of species was undertaken for the collected *Mansonia* and *Uranotaenia* mosquitoes, further studies are needed. There have been historical arboviral outbreaks in Guinea so additional work should be undertaken to characterize vector longevity, anthropophily/zoophily and susceptibility to infection to determine the vectorial capacity for disease transmission in this country [62]. *Toxorhynchites brevipalpis* and *Lt. tigripes* mosquitoes are not vectors of human pathogens but their larvae, together with *Er. intermedius* larvae, play an important role as predators of other mosquito larvae [63]; further investigation looking at larval density should be undertaken in Guinea. Of all the species recorded in this study in Maferinyah sub-prefecture, those identified as *Cx.* cf. *sitiens* were the most abundant. *Culex sitiens* have the ability to survive in brackish water and if these individuals present in Guinea share this characteristic, they may therefore have more options for breeding sites. *Culex sitiens* can travel long distances [60] and the *Cx.* cf. *sitiens* collected in this study were found in all three sites, some 30 km away from the coast where *Cx. sitiens* might be expected to breed [60]. *Anopheles squamosus* and *An. coustani* (*s.l*.) are secondary vectors of malaria and have been shown to be highly anthropophilic [64]. *Anopheles melas* has not historically been classified as an important malaria vector, particularly when coexisting with *An. gambiae* or *An. arabiensis* (major malaria vectors). However, *An. melas* can tolerate brackish water and has been demonstrated to be anthropophilic if there is abundant availability of human hosts [65], so it could play an important role in transmission of malaria in the coastal regions of Guinea. To our knowledge, *Ae. simpsoni* (*s.l*.), *Cx. p. pipiens* and *Er. intermedius* have not been reported in Guinea [14, 15, 18, 19, 21]. The identification of these species, in addition to the potential presence of *Cx. sitiens* (or a very closely related species), further supports the need to undertake regular entomological surveys to determine mosquito species diversity. In the present study, more than 10,000 mosquitoes were collected in 15 days (30 collection intervals) and 20 species were confirmed from a representative subsample, despite the limitation of definitive species confirmation not being possible for certain specimens due to the absence of sufficiently close comparative sequences available on GenBank. Therefore, it is likely that additional species remain to be reported in Guinea and their potential role in transmission of mosquito-borne diseases needs to be evaluated.

## Conclusions

Mosquito surveillance studies often incorporate both adult mosquito traps and HLCs. This study provides evidence for the comparative performance of five different mosquito trap-lure combinations, in comparison with HLCs in Guinea. The five adult traps mainly collected members of the *An. gambiae* complex with opportunistic feeding behaviours, whereas HLCs were shown to preferentially collect anthropophilic species, demonstrating HLCs may still provide the optimal way to collect primary malaria vectors. However, the ST collected the largest number of mosquitoes and also the largest number of different species across the three study sites, indicating it has beneficial properties for mosquito surveillance, in Guinea and similar sites in West Africa, to provide important entomological data on diverse mosquito populations. Due to the damage that this trap causes to the specimens, its performance could be optimised when used in shorter collection intervals and/or when sufficiently protected from adverse weather. This study has shown the importance of combining molecular tools with the morphological identification of specimens to improve entomological studies, revealing the presence of 25 mosquito species in this region of Guinea.

## Supplementary information

**Additional file 1: Table S1.** Coordinates and description of the sampling points in Maferinyah Centre One, Senguelen and Fandie. Latitude and longitude were obtained using GPS (eTrex 10, Garmin). **Table S2.** Number of mosquitoes collected per site, sampling point, time period and trap. Note that 150 collections were performed in total: 50 collections per site (× 3 sites); 30 collections per trap (× 5 traps); 10 collections per sampling point (× 15 sampling points). **Table S3.** PCR assays. Primers, final volumes and conditions of each PCR assay are shown. **Table S4.** Species confirmed by molecular analysis. Sequencing, or a combination of sequencing and species-specific end-point PCR were used to confirm species. A representative specimen from each species is shown, with GenBank accession numbers for sequences generated in this study provided. **Table S5.** Statistical differences between the abundance of mosquitoes captured by the five traps. Table showing the results of the final Generalised Linear Mixed Model: Abundance ~ Site + (1|Point) + Time + Trap for the difference in the abundance of mosquitoes captured by the 5 traps. **Table S6.** Mosquitoes used for molecular identification. Number and proportion of mosquitoes used for molecular ID within each genus (A), each trap and each site (B).

**Additional file 2: Figure S1.** Environmental data. Temperature (a, c, e), relative humidity (b, d, f) and presence of rain (blue drops) in each study site are shown. Graphs represent the temperature and relative humidity in each sampling point (a-e in Maferinyah Centre One, f-j in Senguelen and k-o in Fandie) across 10 collection intervals (5 days and 5 nights) per site. Note that each interval starts with a night collection followed by a day collection except day 10 in Senguelen, which starts with a day collection due to an interval repetition.

## Data Availability

The dataset generated, analysed and supporting the conclusions of this article are available in the OSF repository, https://osf.io/pfma3/.

## Abbreviations

BG2T: BG sentinel 2 trap
BG2-BG: BG sentinel 2 trap with BG lure
BG2-MB5: BG sentinel 2 trap with MB5 lure
GLMM: generalized linear mixed model
GT: Gravid trap
HLC: Human Landing Catch
LT: CDC light trap
ST: Stealth trap
