# Supplementary material for: An assessment of adult mosquito collection techniques for studying species abundance and diversity in Maferinyah, Guinea
Source: Parasit Vectors. 2020 Mar 24;13:150. doi: 10.1186/s13071-020-04023-3 (PMC7092564; doi:10.1186/s13071-020-04023-3)
Supplement: Supplementary file 1 — Additional file 1: Table S1. Coordinates and description of the sampling points in Maferinyah Centre One, Senguelen and Fandie. Latitude and longitude were obtained using GPS (eTrex 10, Garmin). Table S2. Number of mosquitoes collected per site, sampling point, time period and trap. Note that 150 collections were performed in total: 50 collections per site (× 3 sites); 30 collections per trap (× 5 traps); 10 collections per sampling point (× 15 sampling points). Table S3. PCR assays. Primers, final volumes and conditions of each PCR assay are shown. Table S4. Species confirmed by molecular analysis. Sequencing, or a combination of sequencing and species-specific end-point PCR were used to confirm species. A representative specimen from each species is shown, with GenBank accession numbers for sequences generated in this study provided. Table S5. Statistical differences between the abundance of mosquitoes captured by the five traps. Table showing the results of the final Generalised Linear Mixed Model: Abundance ~ Site + (1|Point) + Time + Trap for the difference in the abundance of mosquitoes captured by the 5 traps. Table S6. Mosquitoes used for molecular identification. Number and proportion of mosquitoes used for molecular ID within each genus (A), each trap and each site (B). [file 13071_2020_4023_MOESM1_ESM.docx]

**Additional file 1**

**Table S1.** Coordinates and description of the sampling points in Maferinyah Centre One, Senguelen and Fandie. Latitude and longitude were obtained using GPS (eTrex 10, Garmin).

| Site | Point | Latitude | Longitude | Description |
| --- | --- | --- | --- | --- |
| Maferinyah Centre I (semi-urban) | A | 09.54650 | -013.28160 | Between crops, a rice field and a house. Likely hosts: humans. |
|  | B | 09.54646 | -013.28195 | Behind the house. Likely hosts: humans and goats. |
|  | C | 09.54625 | -013.28157 | In the rice field, under a banana tree. Likely hosts: humans. |
|  | D | 09.54673 | -013.28137 | Far from the house, at the end of the crops. Likely hosts: humans. |
|  | E | 09.54689 | -013.28164 | In front of the house, cooking area. Likely hosts: humans, poultry and cats. |
| Senguelen (rural) | F | 09.41150 | -013.37564 | Close to the road. Likely hosts: goats, chicken and humans. |
|  | G | 09.41117 | -013.37548 | Close to houses. Likely hosts: humans, chicken and goats. |
|  | H | 09.41113 | -013.37511 | Behind the toilet and close to the house. Likely hosts: humans, chicken and goats. |
|  | I | 09.41192 | -013.37514 | Close to a house, under a banana tree. Likely hosts: humans, goats and chicken. |
|  | J | 09.41183 | -013.37552 | The closest to breeding sites and salty water. Close to cooking and resting area, under a banana tree. Likely hosts: humans, goats and chicken. |
| Fandie  (semi-rural) | K | 09.53047 | -013.24000 | Between the rice field and the house. Likely hosts: humans. |
|  | L | 09.53044 | -013.23956 | In a palm tree field, behind the house yard. Likely hosts: unknown. |
|  | M | 09.53026 | -013.23894 | Close to a school (closed for holidays) and to the road. Next to a water container with stagnant water. Likely hosts: goats and occasionally cows. |
|  | N | 09.53084 | -013.23944 | Close to the house, the cooking area and animal shelter. Likely hosts: humans, chicken, goats and dogs. |
|  | O | 09.53088 | -013.23889 | In the crops. Likely hosts: humans. |

**Table S2.** Number of mosquitoes collected per site, sampling point, time period and trap type. In total, 150 collections were performed; 50 collections per site (x3 sites), 30 collections per trap (x5 traps) and 10 collections per sampling point (x15 sampling points).

| Collection number | Site | Point | Time period | Trap | Mosquito abundance |
| --- | --- | --- | --- | --- | --- |
| 1 | Fandie | A | Evening | BG sentinel BG lure | 0 |
| 2 | Fandie | A | Evening | BG sentinel MB5 lure | 1 |
| 3 | Fandie | A | Evening | CDC light trap | 116 |
| 4 | Fandie | A | Evening | Gravid trap | 15 |
| 5 | Fandie | A | Evening | Stealth trap | 817 |
| 6 | Fandie | A | Morning | BG sentinel BG lure | 0 |
| 7 | Fandie | A | Morning | BG sentinel MB5 lure | 0 |
| 8 | Fandie | A | Morning | CDC light trap | 2 |
| 9 | Fandie | A | Morning | Gravid trap | 2 |
| 10 | Fandie | A | Morning | Stealth trap | 3 |
| 11 | Fandie | B | Evening | BG sentinel BG lure | 8 |
| 12 | Fandie | B | Evening | BG sentinel MB5 lure | 0 |
| 13 | Fandie | B | Evening | CDC light trap | 137 |
| 14 | Fandie | B | Evening | Gravid trap | 7 |
| 15 | Fandie | B | Evening | Stealth trap | 201 |
| 16 | Fandie | B | Morning | BG sentinel BG lure | 3 |
| 17 | Fandie | B | Morning | BG sentinel MB5 lure | 3 |
| 18 | Fandie | B | Morning | CDC light trap | 1 |
| 19 | Fandie | B | Morning | Gravid trap | 0 |
| 20 | Fandie | B | Morning | Stealth trap | 3 |
| 21 | Fandie | C | Evening | BG sentinel BG lure | 1 |
| 22 | Fandie | C | Evening | BG sentinel MB5 lure | 3 |
| 23 | Fandie | C | Evening | CDC light trap | 261 |
| 24 | Fandie | C | Evening | Gravid trap | 4 |
| 25 | Fandie | C | Evening | Stealth trap | 1105 |
| 26 | Fandie | C | Morning | BG sentinel BG lure | 5 |
| 27 | Fandie | C | Morning | BG sentinel MB5 lure | 0 |
| 28 | Fandie | C | Morning | CDC light trap | 2 |
| 29 | Fandie | C | Morning | Gravid trap | 2 |
| 30 | Fandie | C | Morning | Stealth trap | 2 |
| 31 | Fandie | D | Evening | BG sentinel BG lure | 12 |
| 32 | Fandie | D | Evening | BG sentinel MB5 lure | 0 |
| 33 | Fandie | D | Evening | CDC light trap | 175 |
| 34 | Fandie | D | Evening | Gravid trap | 12 |
| 35 | Fandie | D | Evening | Stealth trap | 313 |
| 36 | Fandie | D | Morning | BG sentinel BG lure | 0 |
| 37 | Fandie | D | Morning | BG sentinel MB5 lure | 1 |
| 38 | Fandie | D | Morning | CDC light trap | 0 |
| 39 | Fandie | D | Morning | Gravid trap | 2 |
| 40 | Fandie | D | Morning | Stealth trap | 25 |
| 41 | Fandie | E | Evening | BG sentinel BG lure | 9 |
| 42 | Fandie | E | Evening | BG sentinel MB5 lure | 5 |
| 43 | Fandie | E | Evening | CDC light trap | 545 |
| 44 | Fandie | E | Evening | Gravid trap | 4 |
| 45 | Fandie | E | Evening | Stealth trap | 281 |
| 46 | Fandie | E | Morning | BG sentinel BG lure | 1 |
| 47 | Fandie | E | Morning | BG sentinel MB5 lure | 0 |
| 48 | Fandie | E | Morning | CDC light trap | 0 |
| 49 | Fandie | E | Morning | Gravid trap | 3 |
| 50 | Fandie | E | Morning | Stealth trap | 3 |
| 51 | Maferinyah Centre I | F | Evening | BG sentinel BG lure | 10 |
| 52 | Maferinyah Centre I | F | Evening | BG sentinel MB5 lure | 8 |
| 53 | Maferinyah Centre I | F | Evening | CDC light trap | 114 |
| 54 | Maferinyah Centre I | F | Evening | Gravid trap | 32 |
| 55 | Maferinyah Centre I | F | Evening | Stealth trap | 87 |
| 56 | Maferinyah Centre I | F | Morning | BG sentinel BG lure | 2 |
| 57 | Maferinyah Centre I | F | Morning | BG sentinel MB5 lure | 0 |
| 58 | Maferinyah Centre I | F | Morning | CDC light trap | 0 |
| 59 | Maferinyah Centre I | F | Morning | Gravid trap | 6 |
| 60 | Maferinyah Centre I | F | Morning | Stealth trap | 0 |
| 61 | Maferinyah Centre I | G | Evening | BG sentinel BG lure | 2 |
| 62 | Maferinyah Centre I | G | Evening | BG sentinel MB5 lure | 19 |
| 63 | Maferinyah Centre I | G | Evening | CDC light trap | 9 |
| 64 | Maferinyah Centre I | G | Evening | Gravid trap | 27 |
| 65 | Maferinyah Centre I | G | Evening | Stealth trap | 124 |
| 66 | Maferinyah Centre I | G | Morning | BG sentinel BG lure | 1 |
| 67 | Maferinyah Centre I | G | Morning | BG sentinel MB5 lure | 7 |
| 68 | Maferinyah Centre I | G | Morning | CDC light trap | 0 |
| 69 | Maferinyah Centre I | G | Morning | Gravid trap | 2 |
| 70 | Maferinyah Centre I | G | Morning | Stealth trap | 2 |
| 71 | Maferinyah Centre I | H | Evening | BG sentinel BG lure | 0 |
| 72 | Maferinyah Centre I | H | Evening | BG sentinel MB5 lure | 1 |
| 73 | Maferinyah Centre I | H | Evening | CDC light trap | 17 |
| 74 | Maferinyah Centre I | H | Evening | Gravid trap | 37 |
| 75 | Maferinyah Centre I | H | Evening | Stealth trap | 14 |
| 76 | Maferinyah Centre I | H | Morning | BG sentinel BG lure | 3 |
| 77 | Maferinyah Centre I | H | Morning | BG sentinel MB5 lure | 1 |
| 78 | Maferinyah Centre I | H | Morning | CDC light trap | 0 |
| 79 | Maferinyah Centre I | H | Morning | Gravid trap | 3 |
| 80 | Maferinyah Centre I | H | Morning | Stealth trap | 0 |
| 81 | Maferinyah Centre I | I | Evening | BG sentinel BG lure | 2 |
| 82 | Maferinyah Centre I | I | Evening | BG sentinel MB5 lure | 12 |
| 83 | Maferinyah Centre I | I | Evening | CDC light trap | 13 |
| 84 | Maferinyah Centre I | I | Evening | Gravid trap | 27 |
| 85 | Maferinyah Centre I | I | Evening | Stealth trap | 89 |
| 86 | Maferinyah Centre I | I | Morning | BG sentinel BG lure | 7 |
| 87 | Maferinyah Centre I | I | Morning | BG sentinel MB5 lure | 3 |
| 88 | Maferinyah Centre I | I | Morning | CDC light trap | 1 |
| 89 | Maferinyah Centre I | I | Morning | Gravid trap | 2 |
| 90 | Maferinyah Centre I | I | Morning | Stealth trap | 0 |
| 91 | Maferinyah Centre I | J | Evening | BG sentinel BG lure | 0 |
| 92 | Maferinyah Centre I | J | Evening | BG sentinel MB5 lure | 17 |
| 93 | Maferinyah Centre I | J | Evening | CDC light trap | 6 |
| 94 | Maferinyah Centre I | J | Evening | Gravid trap | 21 |
| 95 | Maferinyah Centre I | J | Evening | Stealth trap | 3 |
| 96 | Maferinyah Centre I | J | Morning | BG sentinel BG lure | 0 |
| 97 | Maferinyah Centre I | J | Morning | BG sentinel MB5 lure | 2 |
| 98 | Maferinyah Centre I | J | Morning | CDC light trap | 0 |
| 99 | Maferinyah Centre I | J | Morning | Gravid trap | 0 |
| 100 | Maferinyah Centre I | J | Morning | Stealth trap | 0 |
| 101 | Senguelen | K | Evening | BG sentinel BG lure | 11 |
| 102 | Senguelen | K | Evening | BG sentinel MB5 lure | 61 |
| 103 | Senguelen | K | Evening | CDC light trap | 193 |
| 104 | Senguelen | K | Evening | Gravid trap | 14 |
| 105 | Senguelen | K | Evening | Stealth trap | 339 |
| 106 | Senguelen | K | Morning | BG sentinel BG lure | 14 |
| 107 | Senguelen | K | Morning | BG sentinel MB5 lure | 4 |
| 108 | Senguelen | K | Morning | CDC light trap | 0 |
| 109 | Senguelen | K | Morning | Gravid trap | 3 |
| 110 | Senguelen | K | Morning | Stealth trap | 35 |
| 111 | Senguelen | L | Evening | BG sentinel BG lure | 50 |
| 112 | Senguelen | L | Evening | BG sentinel MB5 lure | 148 |
| 113 | Senguelen | L | Evening | CDC light trap | 64 |
| 114 | Senguelen | L | Evening | Gravid trap | 24 |
| 115 | Senguelen | L | Evening | Stealth trap | 2096 |
| 116 | Senguelen | L | Morning | BG sentinel BG lure | 2 |
| 117 | Senguelen | L | Morning | BG sentinel MB5 lure | 19 |
| 118 | Senguelen | L | Morning | CDC light trap | 15 |
| 119 | Senguelen | L | Morning | Gravid trap | 7 |
| 120 | Senguelen | L | Morning | Stealth trap | 7 |
| 121 | Senguelen | M | Evening | BG sentinel BG lure | 17 |
| 122 | Senguelen | M | Evening | BG sentinel MB5 lure | 28 |
| 123 | Senguelen | M | Evening | CDC light trap | 436 |
| 124 | Senguelen | M | Evening | Gravid trap | 6 |
| 125 | Senguelen | M | Evening | Stealth trap | 416 |
| 126 | Senguelen | M | Morning | BG sentinel BG lure | 2 |
| 127 | Senguelen | M | Morning | BG sentinel MB5 lure | 4 |
| 128 | Senguelen | M | Morning | CDC light trap | 33 |
| 129 | Senguelen | M | Morning | Gravid trap | 3 |
| 130 | Senguelen | M | Morning | Stealth trap | 279 |
| 131 | Senguelen | N | Evening | BG sentinel BG lure | 5 |
| 132 | Senguelen | N | Evening | BG sentinel MB5 lure | 84 |
| 133 | Senguelen | N | Evening | CDC light trap | 278 |
| 134 | Senguelen | N | Evening | Gravid trap | 11 |
| 135 | Senguelen | N | Evening | Stealth trap | 105 |
| 136 | Senguelen | N | Morning | BG sentinel BG lure | 5 |
| 137 | Senguelen | N | Morning | BG sentinel MB5 lure | 5 |
| 138 | Senguelen | N | Morning | CDC light trap | 2 |
| 139 | Senguelen | N | Morning | Gravid trap | 4 |
| 140 | Senguelen | N | Morning | Stealth trap | 2 |
| 141 | Senguelen | O | Evening | BG sentinel BG lure | 36 |
| 142 | Senguelen | O | Evening | BG sentinel MB5 lure | 21 |
| 143 | Senguelen | O | Evening | CDC light trap | 126 |
| 144 | Senguelen | O | Evening | Gravid trap | 14 |
| 145 | Senguelen | O | Evening | Stealth trap | 673 |
| 146 | Senguelen | O | Morning | BG sentinel BG lure | 6 |
| 147 | Senguelen | O | Morning | BG sentinel MB5 lure | 2 |
| 148 | Senguelen | O | Morning | CDC light trap | 2 |
| 149 | Senguelen | O | Morning | Gravid trap | 1 |
| 150 | Senguelen | O | Morning | Stealth trap | 72 |

**Table S3.** PCR assays. Primers, final volumes and conditions of each PCR assay are shown.

| Gene target and reference | Components | Final volume | Conditions |
| --- | --- | --- | --- |
| *ACE*  Smith and Fonseca [29] | 10µL Taq MM 2X  0.2µM pipF (5’-GGAAACAACGACGTATGTACT-3’)  0.4µM quinF (5’-CCTTCTTGAATGGCTGTGGCA-3’)  0.4µM B1246R (5’-TGGAGCCTCCTCTTCACGGC-3’)  2µL gDNA | 20µL | 95ºC – 10’  95ºC – 30’’ *  55ºC – 30’’ *  72ºC – 1’ *  35 cycles  72ºC – 5’ |
| *SINE200*  Santolamazza *et al*. [30] | 10µL Taq MM 2X  1µM S200X6.1-F (5'-TCGCCTTAGACCTTGCGTTA-3')  1µM S200X6.1-R (5'-CGCTTCAAGAATTCGAGATAC-3')  2µL cDNA | 20µL | 94ºC – 10’  94ºC – 30’’ *  54ºC – 30’’ *  72ºC – 1’ *  35 cycles  72ºC – 10’ |
| *IGS*  Scott *et al.* [31] | 10µL Taq MM 2X  1µM UN-F (5’-GTGTGCCCCTTCCTCGATGT-3’)  1µM ME-R (5’-TGACCAACCCACTCCCTTGA-3’)  0.5µM GA-R (5’-CTGGTTTGGTCGGCACGTTT-3’)  2µL cDNA | 20µL | 95ºC – 10’  95ºC – 30’’ *  50ºC – 30’’ *  72ºC – 30’’ *  30 cycles  72ºC – 5’ |
| *COI*  Oshaghi *et al*. [32] | 10µL Taq MM 2X  1µM F (5’-GGTCAACAAATCATAAAGATATTGG-3’)  1µM R (5’-TAAACTTCAGGGTGACCAAAAAATCA-3’)  2µL cDNA | 20µL | 94ºC – 4’  94ºC – 1’ *  55ºC – 1’ *  30 cycles  72ºC – 2’  72ºC – 7’ |
| *ITS2*  Beebe and Saul [33] | 10µL Taq MM 2X  1µM ITS2A (5’-TGTGAACTGCAGGACACAT-3’)  1µM ITS2B (5’-TATGCTTAAATTCAGGGGGT-3’)  2µL cDNA | 20µL | 94ºC – 5’  94ºC – 1’ *  52ºC – 1’ *  72ºC – 2’ *  30 cycles  72ºC – 5’ |
| *COI*  Kumar *et al.* [34] | 10µL Taq MM 2X  1µM F (5’-GGATTTGGAAATTGATTAGTTCCTT-3’)  1µM R (5’-AAAAATTTTAATTCCAGTTGGAACAGC-3’)  2µL cDNA | 25µL | 95ºC – 30’’  95ºC – 30’’ *  45ºC – 1’ *  5 cycles  68ºC – 1’ *  95ºC – 30’’ *  30 cycles  51ºC – 1’  68ºC – 1’  68ºC – 5’ |
| *COI*  Folmer *et al.* [35] | 10µL Taq MM 2X  1µM F (5’-GGTCAACAAATCATAAAGATATTGG-3’)  1µM R (5’-TAAACTTCAGGGTGACCAAAAAATCA-3’)  2µL cDNA | 20µL | 95ºC – 5’  95ºC – 40’’ *  45ºC – 1’ *  5 cycles  72ºC – 90’’ *  95ºC – 40’’ *  30 cycles  51ºC – 1’  72ºC – 90’’  72ºC – 5’ |

*Notes*: *Steps which were run for a certain number of cycles.

**Table S4.** Species confirmed by molecular analysis. Sequencing, or a combination of sequencing and species-specific end-point PCR were used to confirm species. A representative specimen from each species is shown, with GenBank accession numbers for sequences generated in this study provided.

| Sample ID (isolate) | Species  (or closest species) | Sampling location | Collection method | Gene fragment (reference) | GenBank accession number |
| --- | --- | --- | --- | --- | --- |
| FANP52.B3 | *An. coustani* | Fandie | CDC light trap | *ITS-2* (Beebe & Saul) | MN545862 |
| MAFP2.D1 | *An. gambiae* s.s*.* | Maferinyah Centre I | BG sentinel 2 MB5 lure | *ITS-2* (Beebe & Saul) | MN545863 |
| FANP43.B12 | *An. coluzzii* | Fandie | Gravid trap | *ITS-2* (Beebe & Saul) | MN545865 |
| SENP14.H7 | *An. melas* | Senguelen | Gravid trap | *ITS-2* (Beebe & Saul) | MN545865 |
| SENTY.Q | *An. squamosus* | Senguelen | CDC light trap | *COI* (Oshaghi *et al*.) | MN552306 |
| FANP58.D9 | *Lt. tigripes* | Fandie | Stealth trap | *COI* (Kumar *et al.*) | MN552290 |
| MAFP6.C7 | *Cx. watti* | Maferinyah Centre I | CDC light trap | *COI* (Kumar *et al.*) | MN552291 |
| MAFP5.A2 | *Cx. pipiens* | Maferinyah Centre I | BG sentinel 2 MB5 lure | *COI* (Kumar *et al.*) | MN552292 |
| MAFP4.A7 | *Cx. quinquefasciatus* | Maferinyah Centre I | BG sentinel 2 MB5 lure | *COI* (Kumar *et al.*) | MN552293 |
| MAFP5.C5 | *Cx.* cf*. watti* | Maferinyah Centre I | Gravid trap | *COI* (Kumar *et al.*) | MN552294 |
| MAFP8.E9 | *Cx.* cf*. sitiens* | Maferinyah Centre I | CDC light trap | *COI* (Kumar *et al.*; Folmer *et al*.) | MN552295; MN552296 |
| MAFP4.A3 | *Ae. aegypti* | Maferinyah Centre I | BG sentinel 2 MB5 lure | *COI* (Folmer *et al.*) | MN552297 |
| MAFP7.F8 | *Ae. vittatus* | Maferinyah Centre I | Stealth trap | *COI* (Folmer *et al.*) | MN552298 |
| MAFP7.G6 | *Ae. fowleri* | Maferinyah Centre I | Stealth trap | *COI* (Folmer *et al.*) | MN552299 |
| FANP44.D1 | *Ae. cumminsi* | Fandie | BG sentinel 2 BG lure | *COI* (Folmer *et al.*) | MN552300 |
| FANP37.E8 | *Ae. argenteopunctatus* | Fandie | Stealth trap | *COI* (Folmer *et al.*) | MN552301 |
| MAFP6.G8 | *Ae. cf. simpsoni* | Maferinyah Centre I | BG sentinel 2 BG lure | *COI* (Folmer *et al.*) | MN552302 |
| SENP19.A2 | *Ae.* cf*. luteocephalus* | Senguelen | Gravid trap | *COI* (Folmer *et al.*) | MN552303 |
| FANP37.H11 | *Ae.* cf*. denderensis* | Fandie | Gravid trap | *COI* (Folmer *et al.*) | MN552304 |
| SENP11.A3 | *Er. intermedius* | Senguelen | BG sentinel 2 BG lure | *COI* (Folmer *et al.*) | MN552305 |

*Notes*: Where most significant BLAST alignments for query sequences gave maximum identities of 98% or higher with a particular species, with no other species giving similar identities, or where species diagnostic PCRs in combination with sequencing provided confirmation, that species is shown. Where the most significant BLAST alignments gave identities below 98%, indicating the lack of comparative sequences available for confirmation, or where distinction between closely related species wasn’t possible, cf. between the genus and species name denotes the most closely related species providing the most significant BLAST alignment.

**Table S5.** Statistical differences between the abundance of mosquitoes captured by the five traps. Table showing the results of the final Generalised Linear Mixed Model: Abundance ~ Site + (1|Point) + Time + Trap for the difference in the abundance of mosquitoes captured by the 5 traps.

| Trap comparison | Estimate | SE | Z value | P-value |
| --- | --- | --- | --- | --- |
| BG2-MB5 vs. BG2-BG | 0.23444 | 0.35778 | 0.655 | 0.96552 |
| LT vs. BG2-BG | 1.54035 | 0.37001 | 4.163 | < 0.001 *** |
| GT vs. BG2-BG | 0.31106 | 0.36288 | 0.857 | 0.91194 |
| ST vs. BG2-BG | 2.63800 | 0.35738 | 7.382 | < 0.001 *** |
| LT vs. BG2-MB5 | 1.30591 | 0.35856 | 3.642 | 0.00245** |
| GT vs. BG2-MB5 | 0.07662 | 0.35144 | 0.218 | 0.99950 |
| ST vs. BG2-MB5 | 2.40356 | 0.34655 | 6.936 | < 0.001 *** |
| GT vs. LT | -1.22929 | 0.376084 | -3.407 | 0.00588 ** |
| ST vs. LT | 1.09764 | 0.31745 | 3.458 | 0.00492 ** |
| ST vs. GT | 2.32694 | 0.35646 | 6.528 | < 0.001 *** |

**Table S6.** Mosquitoes used for molecular identification. Number and proportion of mosquitoes used for molecular ID within each genus (A), each trap and each site (B).

| 1. Molecular ID by genus | | | |
| --- | --- | --- | --- |
| Genus | | **Collected mosquitoes** | **Mosquitoes with molecular ID [%]** |
| *Anopheles* | | 528 | 249 [47.15] |
| *Aedes* | | 905 | 24 [2.54] |
| *Culex* | | 9088 | 96 [1.03] |
| *Eretmapodites* | | 1 | 1 [100] |
|  | | | |
| 1. Molecular ID by site and trap | | | |
| Site | **Trap** | **Collected mosquitoes** | **Mosquitoes with molecular ID [%]** |
| Fandie | BG2 - BG | 39 | 13 [33.3] |
|  | BG2 - MB5 | 13 | 4 [30.77] |
|  | CDC light trap | 1238 | 16 [1.29] |
|  | Gravid trap | 51 | 3 [5.88] |
|  | Stealth trap | 2753 | 20 [0.73] |
|  | Subtotal [%] | 4094 | 56 [1.37] |
| Maferinyah Centre I | BG2 - BG | 27 | 3 [11.11] |
|  | BG2 - MB5 | 70 | 12 [17.14] |
|  | CDC light trap | 159 | 8 [5.03] |
|  | Gravid trap | 157 | 16 [10.19] |
|  | Stealth trap | 319 | 23 [7.21] |
|  | Subtotal [%] | 732 | 62 [8.47] |
| Senguelen | BG2 - BG | 148 | 27 [18.24] |
|  | BG2 - MB5 | 376 | 77 [20.48] |
|  | CDC light trap | 1149 | 31 [2.7] |
|  | Gravid trap | 87 | 11 [12.64] |
|  | Stealth trap | 4024 | 106 [2.63] |
|  | Subtotal [%] | 5784 | 252 [4.36] |
