# Supplementary material for: An assessment of adult mosquito collection techniques for studying species abundance and diversity in Maferinyah, Guinea
Source: Parasit Vectors. 2020 Mar 24;13:150. doi: 10.1186/s13071-020-04023-3 (PMC7092564; doi:10.1186/s13071-020-04023-3)
Supplement: Supplementary file 2 — Additional file 2: Figure S1. Environmental data. Temperature (a, c, e), relative humidity (b, d, f) and presence of rain (blue drops) in each study site are shown. Graphs represent the temperature and relative humidity in each sampling point (a-e in Maferinyah Centre One, f-j in Senguelen and k-o in Fandie) across 10 collection intervals (5 days and 5 nights) per site. Note that each interval starts with a night collection followed by a day collection except day 10 in Senguelen, which starts with a day collection due to an interval repetition. [file 13071_2020_4023_MOESM2_ESM.docx]

**Additional file 2**


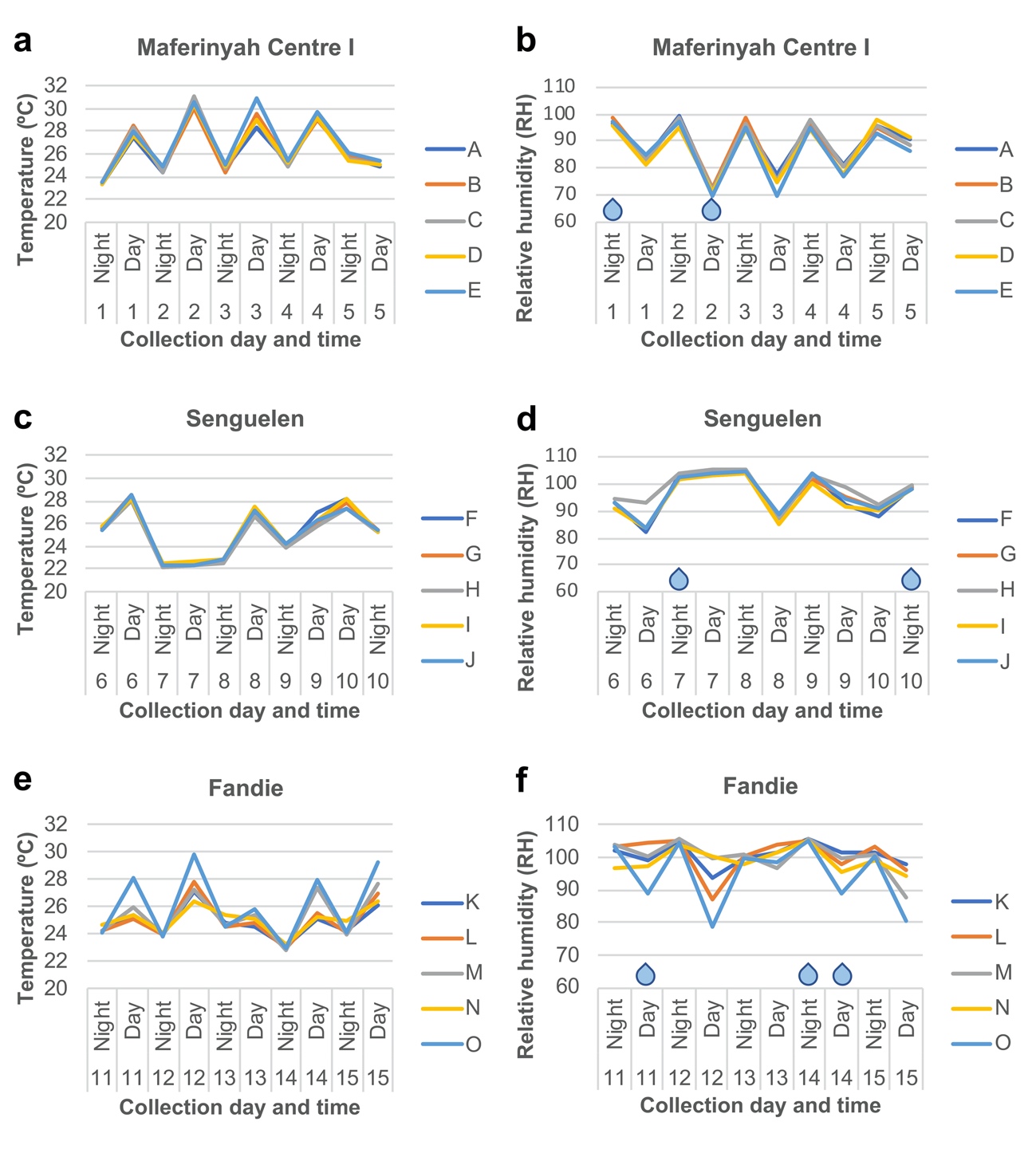


**Figure S1.** Environmental data. Temperature (a, c, e), relative humidity (b, d, f) and presence of rain (blue drops) in each study site are shown. Graphs represent the temperature and relative humidity in each sampling point (a – e in Maferinyah Centre One, f – j in Senguelen and k – o in Fandie) across 10 collection intervals (5 days and 5 nights) per site. Note that each interval starts with a night collection followed by a day collection except day 10 in Senguelen, which starts with a day collection due to an interval repetition.
